# Advances in diet-induced rodent models of metabolically acquired peripheral neuropathy

**DOI:** 10.1242/dmm.049337

**Published:** 2021-11-11

**Authors:** Stéphanie A. Eid, Eva L. Feldman

**Affiliations:** Department of Neurology, School of Medicine, University of Michigan, Ann Arbor, MI 48109, USA

## Abstract

Peripheral neuropathy (PN) is a severe complication that affects over 30% of prediabetic and 60% of type 2 diabetic (T2D) patients. The metabolic syndrome is increasingly recognized as a major driver of PN. However, basic and translational research is needed to understand the mechanisms that contribute to nerve damage. Rodent models of diet-induced obesity, prediabetes, T2D and PN closely resemble the human disease and have proven to be instrumental for the study of PN mechanisms. In this Perspective article, we focus on the development, neurological characterization and dietary fat considerations of diet-induced rodent models of PN. We highlight the importance of investigating sex differences and discuss some of the challenges in translation from bench to bedside, including recapitulating the progressive nature of human PN and modeling neuropathic pain. We emphasize that future research should overcome these challenges in the quest to better mimic human PN in animal models.

## Abstract (precis)

**Summary:** Diet-induced models of peripheral neuropathy highlight the importance of diet composition, duration and source, with mouse strain, age and sex, in recapitulating human disease. Improved models will accelerate translation to the clinic and drug development.

## Introduction

The global prevalence of the metabolic syndrome (MetS; see Glossary, Box 1) has reached epidemic proportions (Saklayen, 2018). Thirty to sixty percent of patients with MetS are affected by peripheral neuropathy (PN; Box 1) (Feldman et al., 2019). For decades, extensive research focused on the role of hyperglycemia in PN development. However, we found that well-controlled glycemia only reduces PN incidence in type 1 diabetes (T1D; Box 1), but marginally improves onset and progression in prediabetes (Box 1) and type 2 diabetes (T2D; Box 1) (Callaghan and Feldman, 2013; Callaghan et al., 2012). Although T2D is the most common cause of PN, central obesity and dyslipidemia are PN risk factors, independent of glycemic status (Andersen et al., 2018; Callaghan et al., 2020). Thus, clinical studies support MetS more broadly as a major PN driver in prediabetes, T2 and obesity, highlighting the metabolically acquired and diet-induced nature of PN (Callaghan et al., 2018, 2016a,b; Christensen et al., 2020). Additionally, guidelines from the American Diabetes Association now recommend lifestyle interventions to improve MetS and prevent PN in prediabetic, T2D and obese patients (www.ada.org) (Pop-Busui et al., 2017).

Although MetS is increasingly recognized as an independent PN risk factor, the cellular and molecular mechanisms underlying disease onset and progression remain unclear. Animal models that closely mimic the human condition have been invaluable in gaining insight into PN pathogenesis. Our laboratory and others have developed rodent models fed a high-fat diet (HFD), which consistently induces MetS, including obesity, impaired glucose tolerance and dyslipidemia, as PN develops (Davidson et al., 2010; Guilford et al., 2011; O'Brien et al., 2020). In this Perspective article, we provide an overview of the guidelines for assessing PN in rodents. We then focus on diet-induced rodent models of obesity, prediabetes and T2D leading to PN, which reproduce human disease and have proven instrumental for studying PN mechanisms. We finally highlight the importance of sex differences and discuss outstanding challenges associated with the use of diet-induced PN models in research.

“Overall, implementing DiaComp recommendations standardizes neuropathy phenotyping, reducing lab-to-lab variation and facilitating the collection of rigorous, reproducible and translatable data, essential for enhancing our understanding of PN.”

**Box 1. Glossary**

**Abnormal sensory symptoms:** frequently experienced by diabetic patients with PN, including allodynia, a pain response to normally innocuous stimuli; hyperalgesia, increased sensitivity to painful stimuli; and/or hypoalgesia, decreased sensitivity to painful stimuli.

**Dorsal root ganglia neurons:** sensory neurons that relay information from the internal and external environments about nociception, touch, temperature or muscle length to the central nervous system.

**Endoneurial microangiopathy:** an abnormality of nerve microvessels including basement membrane thickening and endothelial cell hypertrophy, often accompanying PN development and progression (Fang et al., 2018).

**Hydrogenated vegetable shortening:** a type of fat used in rodent studies. Diets with vegetable shortening can be derived from partially hydrogenated soybean/palm oils or from partially hydrogenated soybean/cottonseed oils (Kubant et al., 2015).

**Hyperphagia:** excessive food intake, which in rodents is induced by a spontaneous mutation in the satiety factor leptin (*ob/ob* mice) or its receptor (*db*/*db* mice or Zucker diabetic fatty rats), leading to obesity and type 2 diabetes (T2D).

**Intraepidermal nerve fiber density (IENFD):** an assessment of small unmyelinated fibers. IENFD is a quantitative approach for the diagnosis of small-fiber neuropathy used in both the clinical and pre-clinical settings (Juster-Switlyk and Smith, 2016).

**Metabolic syndrome (MetS):** a cluster of metabolic risk factors that encompasses elevated fasting glucose (i.e. prediabetes leading to frank T2D), central obesity, dyslipidemia and hypertension (Saklayen, 2018).

**Nerve conduction velocity (NCV):** the speed at which an electrical impulse is transmitted through peripheral nerves. It is the gold standard for PN diagnosis in the clinical and preclinical settings and quantifies the extent of large myelinated nerve fiber dysfunction. NCV studies are reported in m/s and include sensory NCVs measured in the sural nerve following antidromic supramaximal stimulation at the ankle, in turn quantified by dividing the distance by the sensory nerve action potential take-off latency. Motor NCVs in the sciatic nerve are recorded at the foot dorsum following orthodromic supramaximal stimulation, first at the ankle then at the sciatic notch. Sciatic motor NCVs are quantified by subtracting ankle distance from notch distance and dividing by the difference in ankle and notch latencies (Hinder et al., 2017).

**Nociception:** the neurophysiological encoding of actual or potential tissue damage.

**Peripheral neuropathy (PN):** a debilitating degeneration of peripheral nerves in a distal-to-proximal manner, which can lead to chronic pain, non-healing ulcers and lower-limb amputations (Feldman et al., 2019).

**Prediabetes:** characterized by impaired glucose tolerance, often leading to frank T2D. Like T2D patients, prediabetic patients experience long-term complications, including nerve damage or peripheral neuropathy.

**Type 1 diabetes (T1D):** an autoimmune disease characterized by pancreatic β-cell destruction, which leads to insulin deficiency and hyperglycemia (DiMeglio et al., 2018). It accounts for up to 5-10% of all cases of diabetes.

**Type 2 diabetes (T2D):** a component of MetS characterized by hyperglycemia, impaired insulin signaling and dyslipidemia. It is the most common form of diabetes and, in addition to genetic factors, is primarily driven by lifestyle factors such as unhealthy diets and limited physical activity (Chatterjee et al., 2017).

**von Frey filaments:** used to quantify mechanical sensitivity ranging from hyperalgesia or allodynia to lack of sensation or hypoalgesia.

## Guidelines for assessing PN in rodents

**Fig. 1.**
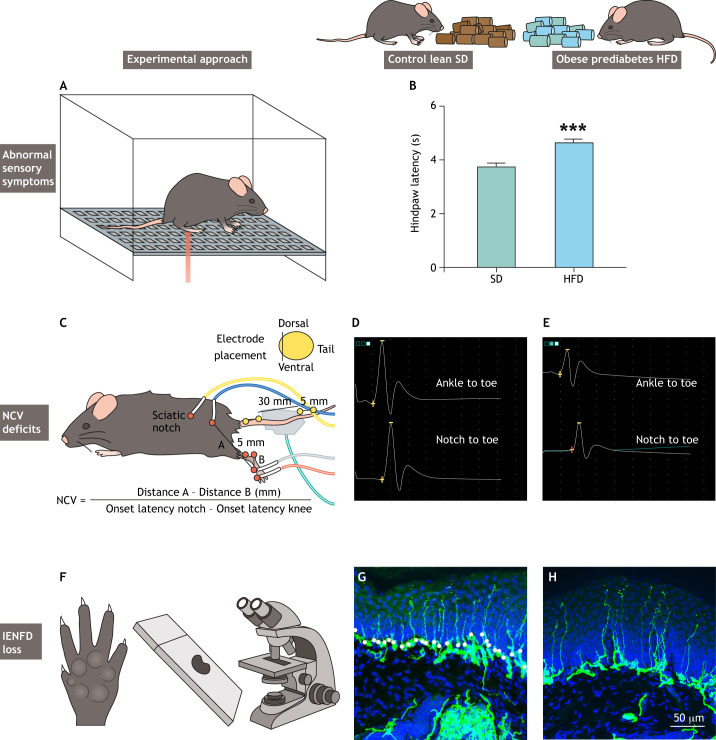
**Neuropathy phenotyping in diet-induced rodent models.** (A) Abnormal sensory symptoms including allodynia, hyperalgesia and/or hypoalgesia are evaluated by testing rodents' sensitivity to a heat stimulus applied to the hindpaw. (B) Typical hindpaw withdrawal latency in 60% high-fat diet (HFD) versus standard diet (SD) mice at 36 weeks (***P<0.001) (reproduced with permission from O'Brien et al., 2018). (C) Electrode placement to record electrophysiological sciatic motor and sural sensory nerve conduction velocities (NCVs) as measures of large nerve fiber impairment [reproduced with permission from protocols.io (dx.doi.org/10.17504/protocols.io.7rbhm2n) under the terms of the Creative Commons Attribution License]. To calculate sciatic motor NCV (m/s), the difference between distance A and distance B (mm) is divided by the difference between the two onset latencies of the compound muscle action potentials (ms). (D,E) Typical sciatic motor traces recorded after stimulation at the ankle and at the notch in a control lean mouse placed on a SD (D) and in an obese prediabetes mouse placed on a HFD (E). (F) Quantifying intraepidermal nerve fiber density (IENFD) in mice footpads serves as histological evidence of small sensory nerve fiber loss. (G,H) Representative images of IENFD in SD (G) and HFD (H) mice at 36 weeks. Scale bar: 50 µm.

Rodent models are useful for studying PN etiology because they facilitate experiments that are not feasible in the clinical setting. The National Institutes of Health created the Diabetic Complications Consortium (DiaComp; www.diacomp.org) to identify new animal PN models and standardize neuropathy phenotyping to reduce lab-to-lab variation. DiaComp advises that a robust rodent PN model should exhibit essential pathological features of the human disease, including abnormal sensory symptoms (Fig. 1A,B; Box 1) such as allodynia, hyperalgesia and/or hypoalgesia; nerve conduction velocity (NCV; Box 1) deficits (Fig. 1C-E); and morphological evidence of intraepidermal nerve fiber density (IENFD; Box 1) loss (Juster-Switlyk and Smith, 2016) (Fig. 1F-H).

Based on DiaComp recommendations, neuropathy phenotyping first assesses thermal sensitivity as a measure of sensory dysfunction using tail-flick or hindpaw withdrawal tests. Alternatively, von Frey filaments (Box 1) can be used to quantitatively assess sensitivity to mechanical stimuli. Like humans, diabetic rodents first develop thermal hypersensitivity and mechanical allodynia followed by decreased sensitivity or hypoalgesia at later disease stages (Feldman et al., 2017). Next, neuropathy phenotyping records electrophysiological sciatic motor and sural sensory NCVs as measures of large nerve fiber impairment (Fig. 1). Lastly, quantifying IENFD in mice footpads serves as histological evidence of small sensory nerve fiber loss (Hinder et al., 2017; Sullivan et al., 2007). These structural changes are paralleled by endoneurial microangiopathy (Box 1) (Fang et al., 2018) and inflammation (Pop-Busui et al., 2016) in peripheral nerves, which can be evaluated to further characterize the animal model. In addition to neuropathy phenotyping, it is important to metabolically profile obese, prediabetic and diabetic rodents, including body weights, glycemic status and insulin levels. Furthermore, our research has shown that dyslipidemia is an independent PN risk factor in obesity, prediabetes and T2D (Eid et al., 2019; O'Brien et al., 2020), and should therefore be examined through a basic lipid profile.

Overall, implementing DiaComp recommendations standardizes neuropathy phenotyping, reducing lab-to-lab variation and facilitating the collection of rigorous, reproducible and translatable data, essential for enhancing our understanding of PN.

## Rodent models of obesity, prediabetes, T2D and PN

Because of their genetic similarities to humans, rodents are considered the model of choice in PN research and have considerably enhanced our understanding of human PN. In addition, they have been useful for evaluating responsiveness to novel or commonly prescribed therapeutic agents, identifying mechanisms of action in peripheral nerves (Eid et al., 2021a,b, 2020). Another advantage is the ability to genetically manipulate genes of interest in PN. Earlier animal studies mostly employed rat PN models (Jakobsen and Lundbaek, 1976; Yagihashi et al., 1979); however, focus has shifted recently to mice PN models (O'Brien et al., 2014) because they are more cost effective and have shorter breeding cycles. Obesity, prediabetes and T2D rodent models of PN include HFD-induced, spontaneous monogenic mutations and polygenic strains. Spontaneous mutations in leptin (*ob*/*ob* mice) or leptin receptor (*db*/*db* mice or Zucker diabetic fatty rats) induce T2D secondary to hyperphagia (Box 1). Although these models consistently develop PN and have been very useful (Eid et al., 2020; O'Brien et al., 2015), they are limited by not adequately modeling human PN progression. Most patients gradually develop hyperglycemia before overt T2D, whereas these T2D rodent models rapidly develop hyperglycemia, largely bypassing the prediabetic stage (Hinder et al., 2018; Oltman et al., 2005). Additionally, loss of leptin signaling may also confound translatability to individuals with prediabetes and T2D by differentially impacting glucose and lipid metabolism irrespective of obesity and T2D (Wang et al., 2014). By contrast, polygenic T2D mouse models gradually develop MetS components, closely mimicking the human disease (O'Brien et al., 2014). Surprisingly, there are no published reports of PN in these polygenic mice, although they hold great promise as potential models mirroring human development of MetS. Therefore, our laboratory is currently examining whether nerve dysfunction occurs in these mice.

Although genetic and spontaneous models are valuable research tools for studying PN, this Perspective article will focus on diet-induced PN models, which have an impressive array of experimental advantages.

## Diet-induced rodent models of obesity, prediabetes, T2D and PN

HFD generates exemplary obesity and prediabetes models with PN. Our laboratory’s clinical findings demonstrate that obesity and prediabetes are major PN drivers (Andersen et al., 2018; Callaghan et al., 2018, 2020, 2016b). Rodents fed increased dietary fat progressively display metabolic disturbances, including weight gain, insulin resistance, dyslipidemia and impaired glucose tolerance in the absence of hyperglycemia. These metabolic changes are often accompanied by compromised responses to stimuli, delayed sensory and/or motor NCVs, and IENFD loss, characteristic of human PN (Guilford et al., 2011; O'Brien et al., 2020).

**Figure:**
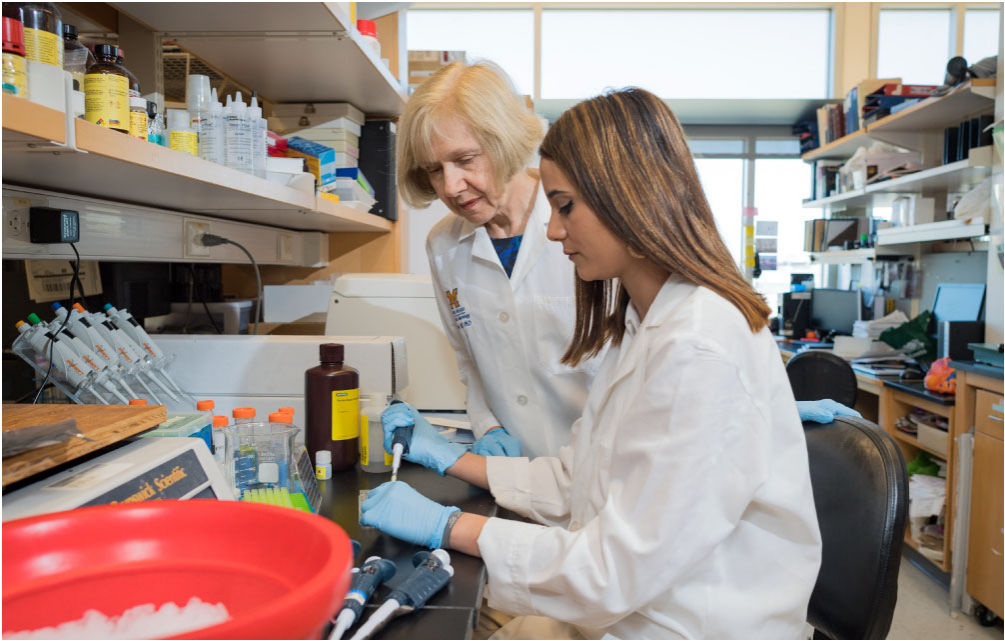
Eva Feldman (back) and Stéphanie Eid (front)

To date, most diet-induced murine studies have used the C57BL/6J strain (Coppey et al., 2018b; O'Brien et al., 2020). However, distinct background strains differentially impact the metabolic and neuropathic phenotypes in response to HFD (Hinder et al., 2017; Sullivan et al., 2007). To identify the optimal mouse strain in HFD-induced obesity, prediabetes and PN, our laboratory recently compared commonly used strains in diabetes research: BKS, BTBR and C57BL/6J. We found that C57BL/6J mice fed a HFD (54% kcal fat) develop the most robust obesity, prediabetes and PN phenotypes (Hinder et al., 2017), advocating it as the strain of choice in diet-induced PN models.

In addition to background strain, rodent age, as well as HFD duration, percentage and dietary fat source, impact the severity of nerve damage in diet-induced models. The percentage of fat content in HFD models can be fine-tuned to optimize the PN phenotype. Earlier studies utilized 42-54% HFD (Table 1); however, our most recent studies using 60% HFD between 11 and 31 weeks indicate that increasing the percentage of dietary fat induces a more pronounced metabolic phenotype, in agreement with The Jackson Laboratory reports (https://www.jax.org/jax-mice-and-services/strain-data-sheet-pages/phenotype-information-380050). Moreover, mice placed on a 60% HFD mice develop severe small- and large-fiber PN after 11 weeks of HFD (Table 1) (O'Brien et al., 2020, 2018). Overall, studies thoroughly comparing the effect of various fat percentages for short to long duration may differentially impact pain, NCVs and IENFDs and must therefore be rigorously accounted for and reported. Such studies will be essential to enhance recapitulation of human PN in animal models.

**Table 1.**
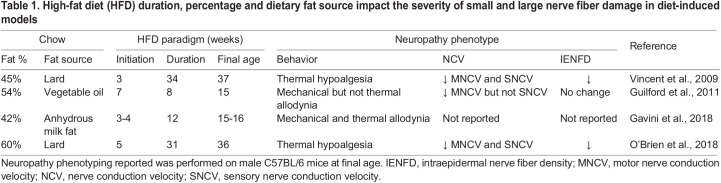
High-fat diet (HFD) duration, percentage and dietary fat source impact the severity of small and large nerve fiber damage in diet-induced models

PN severity also depends on the source of dietary fat and saturation degree (Rumora et al., 2019; Yorek et al., 2017). Lard-derived HFD with 42-60% saturated fats induces obesity, prediabetes and key PN features (Anderson et al., 2014; O'Brien et al., 2020). Conversely, 54% HFD from hydrogenated vegetable shortening (Box 1) does not impair large or small nerve fiber function (Groover et al., 2013). Importantly, replacing lard-based HFD with diets rich in unsaturated fats, such as plant-based and fish oil fats, improves nerve function (Coppey et al., 2018a; Rumora et al., 2019). Our laboratory further shows that supplementing primary dorsal root ganglia neurons (Box 1) *in vitro* with unsaturated fats prevents saturated fat-induced mitochondrial dysfunction (Rumora et al., 2019). Together, these data suggest that fat saturation degree may differentially impact PN progression and that diets rich in unsaturated fats may be neuroprotective, potentially offering an effective lifestyle intervention.

Lastly, ketogenic diets consisting of 79-90% kcal plant-based fat, 8-9% protein and 0.3-3% carbohydrate improved nociception (Box 1) and increased IENFD without reversing pre-existing metabolic abnormalities (Cooper et al., 2018a). How a ketogenic diet improves nerve function remains unclear. However, it is thought that ketone bodies may promote axon growth (Cooper et al., 2018a) and reduce inflammation (Ruskin et al., 2009). These results suggest that ketogenic strategies may be promising in the treatment of metabolically acquired PN and should therefore be validated in the future.

“A better understanding of sex dimorphism in PN is critical for tailoring sex-specific therapeutic strategies.”

## Sex differences in rodent models of PN

Sex differences are evident in the prevalence, clinical manifestations and etiology of MetS in humans (Pradhan, 2014) and differential responsiveness to antidiabetic drugs (Franconi and Campesi, 2014). There is also increased prevalence of MetS postmenopause in female individuals, presumably due to estrogen deficiency (Lovre et al., 2016). However, clinical studies investigating sex as a potential differential PN risk factor are limited and inconclusive. One study reported that male T2D individuals were affected by PN earlier than females (Aaberg et al., 2008), which mirrors recent findings that female T2D individuals experience more frequent and intense neuropathic pain despite a milder PN phenotype (Abraham et al., 2018). However, larger prospective cohorts are required to validate these findings. Importantly, a better understanding of sex dimorphism in PN is critical for tailoring sex-specific therapeutic strategies. This view is in accordance with a recent National Institutes of Health policy, which now requires preclinical studies to account for sex differences, reflecting efforts to be more inclusive of unrepresented groups in clinical studies (Clayton and Collins, 2014).

In this vein, animal studies, which previously focused heavily on male rodents, are starting to investigate the role of sex dimorphism in obesity and prediabetes (Zore et al., 2018). Male rodents, such as C57BL/6J mice, are more susceptible to diet-induced obesity and prediabetes versus female littermates (Casimiro et al., 2021; Hwang et al., 2010; Yang et al., 2014). Additionally, inducing obesity and glucose intolerance is more challenging in female Sprague-Dawley rats, requiring a higher dietary fat percentage and longer feeding duration relative to males (Coppey et al., 2018b). In line with an earlier report (Pettersson et al., 2012), our laboratory recently showed that HFD female mice are protected from insulin resistance early during HFD feeding versus male littermates (Elzinga et al., 2021). Interestingly, as in humans (Janssen et al., 2008; Lovre et al., 2016), estrogen exerts antidiabetic effects on MetS and T2D in rodents (Riant et al., 2009). Several mechanisms may contribute to these protective actions, including regulation of pro-inflammatory mediators and lipid metabolism (Bryzgalova et al., 2008; Stubbins et al., 2012), which are both crucial for nerve health (Mitro et al., 2017; Pop-Busui et al., 2016). There are clear differences between male and female obesity and prediabetes rodent models, which could have clinical diagnostic and management implications.

Although female mice are clearly protected from metabolic dysfunction, at least early in the disease course, sex-specific effects on PN are much less clear-cut. Our laboratory and others are addressing this issue by characterizing PN in two common animal models, C57BL/6J mice (Elzinga et al., 2021; Obrosova et al., 2007) and Sprague-Dawley rats (Coppey et al., 2018b). Our results in 60% HFD mice indicate that females develop PN to the same degree as males, despite early protection against insulin resistance (Elzinga et al., 2021), similar to findings in HFD-fed C57BL/6J mice and Sprague-Dawley rats that were treated with low-dose streptozotocin to generate a T2D model (Coppey et al., 2018b). Overall, these reports evaluated PN at a relatively late disease stage, and future studies investigating sex differences during the early stages of PN development are critical. Hormone levels may differentially affect the metabolic and neuropathic phenotypes; thus, it is also important to assess sex hormones in future studies.

Why female rodents still develop PN in obesity, prediabetes and T2D, despite a ‘healthier’ metabolic profile, at least early in the study, is unclear. Peripheral nerves in female mice still accumulate sorbitol and oxidative stress and exhibit poly (ADP-ribose) polymerase activation (Obrosova et al., 2007). Interestingly, these pathways mediate, at least in part, PN in male neuropathic mice (Feldman et al., 2017) and may be implicated in disease development in female mice. Our transcriptomic analysis of peripheral nerves from *ob/ob* mice identified dysregulation of inflammatory and immune response pathways in female mice (O'Brien et al., 2016), similar to our findings in peripheral nerve tissue from male mice (O'Brien et al., 2015). Although these results suggest similar mechanisms of nerve injury, regardless of sex, further research is required to define sex differences in PN, in rodents and humans, to develop effective disease-modifying therapies in both sexes, or tailor sex-specific therapies.

## Challenges translating from bench-to-bedside

The limitations of diet-induced rodent models in accurately mimicking the metabolic aspect of human obesity and T2D have been previously laid out (Lai et al., 2014). In this section, we focus on challenges associated with modeling human PN.

### Does diet-induced PN in rodents faithfully recapitulate gradual disease onset in humans?

The pathogenesis of prediabetes, T2D and PN in humans is driven by a complex interaction of environmental and genetic factors resulting in gradual disease progression over a long period of time (Wysham and Shubrook, 2020). Conversely, HFD rodent studies are often initiated at a young age roughly equivalent to that of a human teenager, as per The Jackson Laboratory reports (https://www.jax.org/news-and-insights/jax-blog/2017/november/when-are-mice-considered-old#:~:text=Mature%20adult%20mice%20range%20in,ranges%20from%2020%20%2D%2030%20years). Within only 4 weeks of diet, they display components of the MetS, including obesity and insulin resistance, although to variable extents (Wang and Liao, 2012). Similarly, HFD mice, within this 4-week timeframe, start to exhibit PN features, such as mechanical hypersensitivity (Groover et al., 2013), and an established PN phenotype is observed by 8 weeks of HFD (Cooper et al., 2018b; Guilford et al., 2011). Subsequently, the duration of most diet-induced rodent studies in PN research is often short, at a few weeks to a few months at most, which is roughly equivalent to human adult age (O'Brien et al., 2014) and therefore does not fully reflect the progressive nature of human PN. This is particularly true regarding neuropathic pain (discussed in detail below), which is commonly experienced by prediabetic and T2D patients (Abbott et al., 2011) and not accurately recapitulated in diet-induced models. Hence, these findings can potentially lead to inaccurate depiction of disease pathogenesis in humans, which should be validated in other robust PN models and clinical studies. This also applies to drug studies that are effective in diet-induced rodent models, but do not necessarily translate to improved outcomes for prediabetic and T2D patients.

Another translational roadblock is the more variable PN phenotype in humans versus a more uniform phenotype in rodents. As mentioned earlier, most rodent studies consist of same-sex animals on the same genetic background, which likely generate a similar level of nerve dysfunction. By contrast, distinct lifestyle factors and genetic predisposition in humans likely lead to variable PN presentation across prediabetic and T2D individuals (Feldman et al., 2019). Studies including different background strains within a single animal cohort are therefore essential to mimic patients with varying susceptibilities to metabolic disorders and PN.

“In light of the current obesity pandemic, increased preclinical knowledge from diet-induced rodents will accelerate the development of disease-modifying therapies for treating metabolically induced neuropathic pain, which remains largely understudied.”

### Neuropathic pain

Neuropathic pain is a common, and often the earliest, consequence of PN, affecting up to 50% of diabetic patients (Abbott et al., 2011). It manifests as hypersensitivity with hyperalgesia/allodynia or as spontaneous and continuous pain sensations (Feldman et al., 2017). Neuropathic pain is often accompanied by disturbed sleep, anxiety and depression, which reduce patients' quality of life (Sloan et al., 2018). Unfortunately, current therapies have limited efficacy due to incomplete understanding of the pathophysiological mechanisms of pain. Diabetic animal models, especially streptozotocin-induced T1D rodents, have been pivotal for studying pain processing and testing candidate therapeutics (Jaggi et al., 2011). However, much less is known on the interplay between components of MetS and neuropathic pain, mostly due to lack of an established animal model that consistently develops pain symptoms. Two recent studies reported contradictory findings regarding pain response in the commonly used C57BL/6J HFD mouse. One study examined the effect of genetic differences among C57BL/6 mice from two different suppliers on pain behaviors following a 7-week plant-based HFD, observing that C57BL/6 mice from The Jackson Laboratory retained normal mechanical sensitivity, whereas C57BL/6 mice from Charles River Laboratories displayed pain behaviors (Cooper et al., 2018b). By contrast, another study observed that administering a Western diet containing 42% kcal from anhydrous milkfat for 12 weeks induced mechanical allodynia and thermal hyperalgesia in C57BL/6 mice from The Jackson Laboratory (Bonomo et al., 2020). In addition to different diet duration, a key difference between the two reports is dietary composition, particularly the high-sucrose (34%) and -cholesterol (0.2%) content of the Western diet, key drivers of MetS, which could account for the discrepancies in pain behaviors. Standardizing paradigms used to induce MetS, including HFD composition and duration, are required to establish a rodent model with reproducible and consistent pain phenotype, which should be a future avenue of work. Another critical consideration is including older animals to longitudinally assess pain behaviors that start early during HFD. As emphasized earlier, determining sex-specific pain responses is also key, especially because human studies show that T2D female individuals are more prone to pain than males (Abraham et al., 2018). In light of the current obesity pandemic, increased preclinical knowledge from diet-induced rodents will accelerate the development of disease-modifying therapies for treating metabolically induced neuropathic pain, which remains largely understudied.

## Conclusions

PN is a frequent and complex complication of obesity, prediabetes and T2D, which requires better understanding to progress translational research and develop much needed mechanism-based therapies. Herein, we focused on diet-induced rodent models, which, although not perfect, share essential metabolic and neurological features with human obesity, prediabetes, T2D and PN. We, therefore, recommend them to researchers investigating nerve damage induced by MetS. In light of the growing obesity pandemic, diet-induced rodent studies have emphasized the importance of dietary interventions as a treatment for PN, in agreement with the American Diabetes Association recommendations. Moving forward, research should address the mechanisms underlying the beneficial effects of dietary interventions on PN, which will help inform the optimal dietary regimen and develop targeted therapies for PN patients unable or unwilling to engage in changes in dietary habits.
